# Green Procedure to Manufacture Nanoparticle-Decorated Paper Substrates

**DOI:** 10.3390/ma11122412

**Published:** 2018-11-29

**Authors:** Werner Schlemmer, Wolfgang Fischer, Armin Zankel, Tomislava Vukušić, Gregor Filipič, Andrea Jurov, Damjan Blažeka, Walter Goessler, Wolfgang Bauer, Stefan Spirk, Nikša Krstulović

**Affiliations:** 1 Institute of Paper-, Pulp- and Fibre Technology (IPZ), Graz University of Technology, Inffeldgasse 23, 8010 Graz, Austria; werner.schlemmer@gmx.at (W.S.); wolfgang.johann.fischer@gmail.com (W.F.); wolfgang.bauer@tugraz.at (W.B.); 2 Institute of Electron Microscopy and Nanoanalysis (FELMI), Steyrergasse 17, 8010 Graz, Austria; armin.zankel@felmi-zfe.at; 3 Faculty of Food Technology and Biotechnology, University of Zagreb, Pierottijeva 6, 10000 Zagreb, Croatia; tvukusic@pbf.hr; 4 Jožef Stefan Institute, Jamova 39, Ljubljana 1000, Slovenia; Gregor.Filipic@ijs.si (G.F.); andrea.jurov@ijs.si (A.J.); 5 Jožef Stefan International Postgraduate School, Jamova 39, Ljubljana 1000, Slovenia; 6 Institute of Physics, Bijenička 46, 10000 Zagreb, Croatia; dblazeka@ifs.hr; 7 Institute of Chemistry, University of Graz, Universitaetsplatz 1, 8010 Graz, Austria; walter.goessler@uni-graz.at

**Keywords:** silver nanoparticles, laser ablation in liquids, laser synthesis of colloidal nanoparticles solution, nanoparticle-impregnated paper, antimicrobial activity, fiber fines, sheet forming, vacuum filtration

## Abstract

For this study, a paper impregnated with silver nanoparticles (AgNPs) was prepared. To prepare the substrates, aqueous suspensions of pulp fines, a side product from the paper production, were mixed with AgNP suspensions. The nanoparticle (NP) synthesis was then carried out via laser ablation of pure Ag in water. After the sheet formation process, the leaching of the AgNPs was determined to be low while the sheets exhibited antimicrobial activity toward Escherichia coli (*E. coli*).

## 1. Introduction

Cellulose, a biopolymer consisting of β-D 1→4 linked glucose, is the main component in many industrial products such as pulp and paper. In recent years, the development of micro- and nanostructured materials (e.g., nanocellulose and microfibrillated cellulose) was a boost to cellulose science and new applications for industrial purposes have been proposed. One way to create the new materials is to include inorganic matter having particular properties. In this context, the incorporation of functional inorganic nanoparticles (NPs) (e.g., antimicrobial, conductive, photoactive, catalytic, magnetic, Raman active) into different types of cellulosic substrates has been described for many cellulosic materials [1,2,3,4,5,6,7,8,9]. In principle, there are different ways of implementing nanoparticles into these materials. One approach is the so called in situ synthesis, where the nanoparticle precursor, typically a metal salt or metal citrate, is added to a cellulosic material. The metal ions coordinate to the cellulose macromolecules and a chemical reaction is induced to convert the salt into either metal, metal oxide, or metal sulfide nanoparticles, which are formed in close spatial proximity to the cellulose material [10]. In this way, silver and gold nanoparticle-decorated fibers have been prepared from AgNO_3_ and HAuCl_4_, in the presence of different types of reduction agents [11,12]. While NaBH_4_ was frequently used in older studies to reduce the metal salts, in recent years the use of environmentally friendly reduction agents has become more popular. Here, either glucose or polysaccharides with reducing end groups (i.e., aldehydes) were used to generate silver and gold nanoparticles [13,14,15,16,17]. In particular, polysaccharides are interesting since they not only act as reduction agents but also serve as electrosteric stabilizers, preventing the aggregation of the nanoparticles and providing suspensions which can be stable for several months [18]. In addition, monodisperse silver nanoparticles (AgNPs) can be prepared using a pulsed sono-electrochemical technique from silver citrate where poly (vinyl pyrrolidone) can serve as a stabilizer [19].

A different route involves the application of the ready-made nanoparticles to a cellulosic material, and then to perform a processing step. If the nanoparticles are stabilized by a polymer shell, covalent or physical binding onto the cellulosic substrate can be additionally performed. For example, silver and gold nanoparticles have been synthesized and encapsulated by sulfated chitosan showing antithrombogenic behavior [3,20]. After immobilization on surfaces, simultaneous antimicrobial and anticoagulant surfaces were obtained. 

The common element in these methods is that a metal salt precursor is required which must be converted to the desired nanoparticles, very often using elevated temperatures that give a particular type of nanoparticle shape (e.g., spheres, rods). In most cases, nanoparticles contain either capping agents or polymer shells to prevent agglomeration and to obtain stable colloidal suspensions. However, the largest drawback of many wet-chemical routes is their yield which is hardly ever reported in scientific papers. In order to overcome limitations and to make the production of nanoparticles scalable and environmentally friendly, laser ablation in liquids (LAL) has been proposed recently for nanoparticle preparation [21]. This technique is based on the process of pulsed laser ablation of a target (metals, metal oxides, nitrides, etc.) immersed in a liquid [22,23,24]. There are many advantages of adopting this technique as compared to standard ones, but its potential has still not been fully exploited. It is known as a “green synthesis” technique since additional chemicals are not required [25] while the formation of any by-products is prevented. In principle, pure nanoparticles can be obtained that do not contain any residues on the surface, except oxidation products (e.g., oxides). The LAL technique is not limited by the choice of materials because any metal target or target made of other materials (composites, isolators, conductive materials, semiconductors, organic materials, ceramics, catalytic, hybrid materials, and magnetic or paramagnetic materials) can be used for the synthesis of colloidal nanoparticle suspensions. Therefore, a wide variety of liquids can be used for tailoring the nanoparticle properties [26,27]. Laser pulses can additionally generate, de-agglomerate, fragmentate, re-shape, and reduce the size of the initially formed nanoparticles either by secondary laser interaction (post-irradiation) [28,29,30,31] or by double-pulse LAL [32,33,34].

In this paper, the authors explored the use of LAL for preparing AgNPs in water and integrating them into a paper-based substrate, consisting of fine cellulosic microparticles. Sheets from such particles resemble to a significant extent those made of microfibrillated cellulose, a recently proposed material for many applications such as barrier coatings and packaging materials. However, the price of the material is much lower since it is a large-scale underutilized material in cellulose processing industries. The outline of the paper is as follows: after a description of the synthesis/characterization of the NPs by laser ablation, the procedure to incorporate nanoparticles into sheets made from fines is described. Finally, the properties of the substrates in terms of antimicrobial activity as well as the leaching of the AgNPs from the material are evaluated.

## 2. Materials and Methods 

### 2.1. Laser Synthesis of Nanoparticles

A colloidal solution of AgNPs was synthesized using pulsed laser ablation of a Ag plate (purity 99.99%, thickness 1 mm, Kurt J. Lesker, Jefferson Hills, PA, USA) immersed in a cuvette filled with 25 mL of deionized water. Laser ablation was conducted with a Nd:YAG laser (Quantell, Brilliant, Les Ulis, France) using the following specifications: pulse duration 5 ns, wavelength 1064 nm, output energy 290 mJ, and repetition rate 5 Hz. The target was fixed on the holder in order to drill craters during ablation. The laser beam was focused by a 10 cm lens onto the target surface. The laser pulse energy in front of the target was 120 mJ while the diameter of a focused pulse on the target surface was 1 mm yielding a laser fluence of 15 J/cm^2^. The thickness of a water layer above the target was kept constant at 1.5 cm during the experiment in order to keep the ablation efficiency constant [35]. A detailed scheme of the experimental setup for laser ablation in water is shown in [36].

The morphology of AgNPs was observed using a transmission electron microscope (TEM, FEI Tecnai G2 20 Twin, Thermo Fischer Scientific, Waltham, MA, USA). Obtained TEM pictures were used to calculate size distribution of the AgNPs. 

After the ablation, the crater created on the Ag target was studied by an optical microscope (Leitz, Leica Aristomet, reflective illumination mode, Wetzlar, Germany) in order to determine the crater’s volume for the assessment of nanoparticle concentration in a procedure described in [37]. The procedure regarding the crater volume determination is described in detail in [38]. It was found that an ablation volume of 23.8·10^6^ μm^3^ yielded a 250 μg mass of ablated material. Under the assumption that most of the ablated material is transferred into nanoparticles, its mass is also the total mass of synthesized nanoparticles. Therefore, the production rate of nanoparticles is 10 μg per mL as ablation is performed in 25 mL of water. From the known size distribution and the crater volume, the concentration of AgNPs is determined to be in the order of 10^10^ mL^−1^.

A colloidal solution of laser-synthesized AgNPs was analyzed using a spectrophotometer (Perkin Elmer, Lambda 25, Waltham, MA, USA) to assess the UV-Vis absorption spectrum. Zeta-potential (Zetasizer, Malvern Instruments, Worcestershire, Great Britain) of Ag colloidal solution was measured as −50 mV, indicating a colloidal solution of high stability (the solution is stable for months). 

AgNP-impregnated fines sheets were analyzed by scanning electron microscope (SEM, Jeol JSM-7600F, Tokyo, Japan). The electron accelerating voltage was set to 10 kV. Before the imaging, samples were coated with a thin layer of amorphous carbon (PECS 682) to prevent charge accumulation on the surface. Observation mode was set to low-energy secondary electron detection. Energy-dispersive X-ray spectroscopy (EDS) was done on the same instrument by an INCA Oxford 350 EDS SDD detector with an accelerated potential of 15 kV.

### 2.2. Hand Sheet Formation

**Figure 1:**
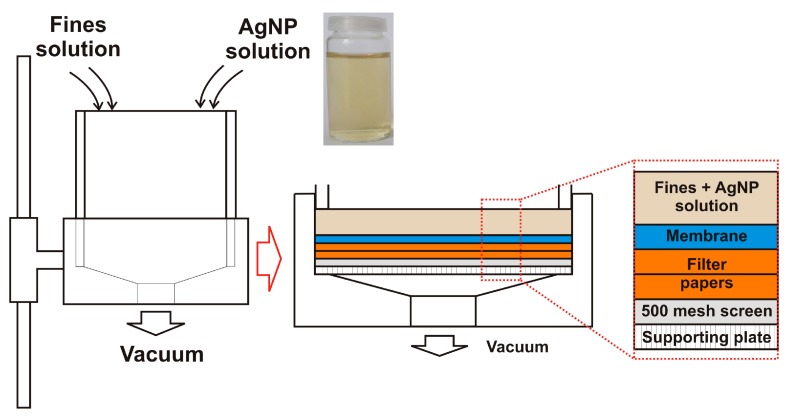
Scheme of the manufacturing process of preparing silver nanoparticle (AgNP)-impregnated sheets.

All tests were performed using primary fines separated from never-dried, bleached sulfite pulp (mixture of spruce and beech). In order to prepare hand sheets from pure fines, a vacuum filtration method described in [39] was used. A defined amount of fines (0.24 g, dry weight) was diluted with deionized water to reach a solid content of 0.24 wt %. This suspension was stirred at 450 rpm for at least 2 h. After stirring, 2 mL of AgNP colloidal solution (10^10^ particles mL^−1^) was added to the suspension and stirred for 5 min. After the addition of AgNPs, the sheets were formed by vacuum filtration using a Britt Dynamic Drainage Jar (Frank PTI, Birkenau, Germany). The scheme used for AgNP-impregnated sheet forming is shown in Figure 1. 

The Britt Dynamic Drainage Jar was equipped with a supporting plate, a 500 mesh screen (hole diameter 20 m), two filter papers, and a nitrocellulose membrane (DAWP29325 from Merck Chemicals and Life Science GesmbH, Darmstadt, Germany) with a pore size of 0.65 m. The major advantage of using the Britt Dynamic Drainage Jar as compared to a Büchner funnel was to improve the fines sheet formation. In particular, the sandwich-like setup prevented the loss of fine cellulosic material. After the filtration step, the membrane with the fines/MFC sheet on top was pre-dried in a Rapid-Köthen sheet dryer (Frank PTI, Birkenau, Germany) for about 20 s at 93 °C under vacuum. Then, the membrane was peeled off, and the neat fines/MFC sheets were dried for 10 min in the Rapid-Köthen sheet dryer. The sheets were then stored in a climate room at 23 °C and 50% RH for least 12 h prior to testing.

From the known concentration of AgNPs, volume of colloidal AgNPs added to the suspension, and sheet dimension (10 cm in diameter), the density of impregnated AgNPs could be calculated. The number density of impregnated AgNPs obtained was in the order of 10 nanoparticles per μm^2^. 

### 2.3. Antimicrobial Test

For the determination of antimicrobial activity, *E. coli* MG1655-K12 was used (donated by the Laboratory for Biology and Microbial Genetics, Faculty of Food Technology and Biotechnology, University of Zagreb, Zagreb, Croatia). The bacterial suspension was prepared by inoculating 20 μL of *E. coli* K12 in 10 mL of nutrient broth (Biolife, Milan, Italy). This bacterial suspension was incubated at 37 °C for 24 h to create bacteria in the stationary growth phase. The incubated bacterial suspension was centrifuged (Tehtnica, Centric 150, Domel, Železniki, Slovenia) at 4000 rpm for 10 min at room temperature. Harvested cells were washed three times and re-suspended in phosphate buffer saline (PBS) and sterile water solution.

Paper impregnated with AgNPs was inoculated using 10–100 μL of the selected microorganism and placed in an Eppendorf tube containing phosphate buffer. The aliquot sample was incubated 0 h, 1 h, 4 h, 6 h, and 24 h, respectively, after the addition of *E. coli* K12 at a temperature of 37 °C. After incubation, the number of microorganisms (CFU/mL) was determined by the standard dilution method on nutrient agar (Biolife, Milan, Italy). As a control, the selected microorganism (100 μL) was inoculated into 900 μL of phosphate buffer, incubated, and counted by the number of augmented cells. All experiments were analyzed three times (as the repetition of three experiments) and the final results were the mean values of three determinations. The results were reported as log colony forming units per milliliter (log CFU/mL).

### 2.4. Leaching Tests and Analysis of the Solutions

To investigate the leaching of AgNPs upon contact with water, the paper substrates were shaken in ultrapure water and samples of the liquid were taken after 5 min, 10 min, 30 min, 1 h, 2 h, 5 h, 8 h, and 24 h. The total Ag concentration in these extracts was determined after acidification with nitric acid (10% v/v) with an Agilent 7700cx ICPMS at m/z 107. National Institute of Standards and Technology (NIST) validated Standard Reference Materials (SRM) including 1640a “Trace elements in water” was used for quality control. The Ag concentration of each extract was determined from three parallel measurements. From these results, the extracted amount of Ag in μg was calculated taking the extraction volume into account. The amount of leached mass was directly calculated from the inductively coupled plasma mass spectrometry (ICP-MS) measurements, whereas the relative leaching was determined with respect to the initially added amount of colloidal AgNPs (1 or 2 mL which corresponded to the addition of 10 or 20 μg of AgNPs, respectively).

## 3. Results

AgNPs feature surface plasmons (SP) activated by the interaction of conductive electrons and an external electromagnetic field, that is, via irradiation with light. If these photons have the right frequency (i.e., the plasmonic frequency), the SPs are excited into a resonant state, oscillating with the highest possible amplitude. This plasmonic frequency depends on the size, shape, chemical composition, and environment of the NPs. From the frequency (wavelength) dependency on the intensity (i.e., absorbance) of the colloidal NPs, which can be assessed by UV-Vis spectroscopy, one can roughly estimate the NP size in solution. 

**Figure 2:**
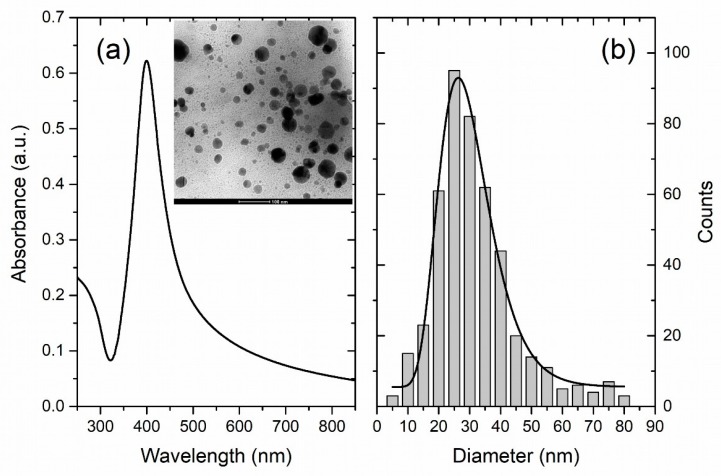
(**a**) UV-Vis photoabsorption spectrum of colloidal AgNPs (Inset: TEM image of AgNPs) and (**b**) Size distribution of AgNPs obtained by TEM imaging (black line: Lognormal fit).

The UV-Vis spectrum and the size distribution of the LAL-synthesized AgNPs are shown in Figure 2a,b. The UV-Vis spectrum of the AgNPs synthesized by laser ablation shows a distinct maximum at 400 nm, typical for a surface plasmon resonance of Ag nanoparticles with a dimension of a few tens of nanometers [10]. This rough estimation is further supported by TEM analysis (inset of Figure 2a), which, in addition, reveals that the AgNPs have a spherical shape. The AgNPs feature an average dimension of 28 nm and their size distribution is relatively broad (FWHM of 20 nm; fitting by a log-normal function (black line)). The LogN fit is often used to describe the size distribution of nanoparticles synthesized from the gaseous phase which is the case in the nanoparticle synthesis process using LAL. It applies whenever particle growth depends on diffusion and drift of atoms to a growth zone of nanoparticles [40]. The final distribution is determined by the available growth time of the nanoparticles [41]. The formation of AgNPs synthesized by LAL is described by dynamic formation mechanisms which include a diffusion slow-growth (diffusion coalescence) process [42,43,44].

After characterization, these nanoparticles were implemented into a sheet forming process. Suspensions containing the AgNPs and the paper fines were mixed and a stable colloidal suspension was obtained. This suspension was then applied to a Britt Jar process in order to avoid any loss of fine cellulosic material as described in [38]. Afterwards, the sheets were transferred to a drying system and hand sheets were obtained. 

**Figure 3:**
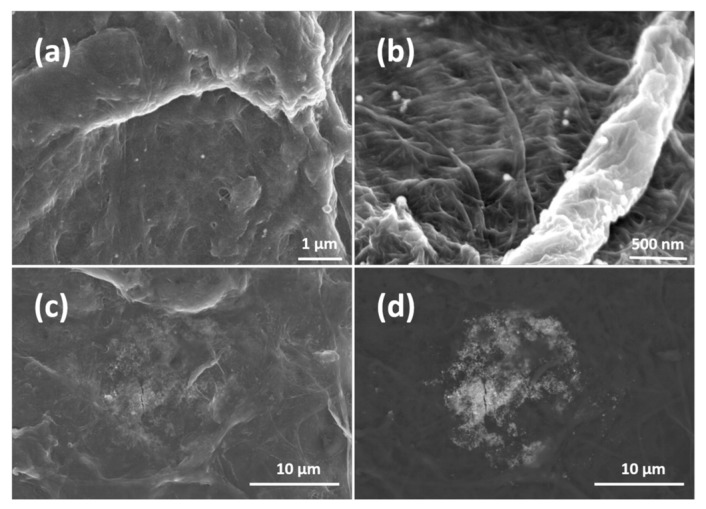
(**a**,**b**) are SEM images of AgNP-impregnated sheets at different magnifications. (**c**,**d**) are the same images recorded with different detectors to show impregnated agglomerates.

Figure 3 depicts SEM images of such AgNP-impregnated hand sheets at different magnification. In Figure 3a,b, distinct AgNPs located at the surface of the sheets can be observed. Identification of the AgNPs was further performed by EDS measurements (shown in the Supplementary Material). Figure 3c,d depicts the same images but recorded with different detectors. They reveal AgNP agglomerates (which are very rare) embedded beneath the sheet surface, covered with much smaller fines fibers. AgNPs in (a) and (b) are in direct contact, whereas those in (c) and (d) are in close contact with bacteria in antimicrobial testing experiments. In Figure 3a,b, one can identify only a few AgNPs per μm^2^ on the sheet surface. It implies that AgNPs are impregnated in the whole volume of the sheet as their density is around 10 AgNPs per μm^2^. The authors assume that AgNPs are impregnated into sheets by physical entrapment. It can be clearly seen that the AgNPs are hardly agglomerated, which relates to the processing conditions. There, the AgNP colloidal suspensions are added to the fines suspensions under rigorous stirring which ensures an even distribution of the AgNPs in the fines suspension and consequently in the formed sheets. 

**Figure 4:**
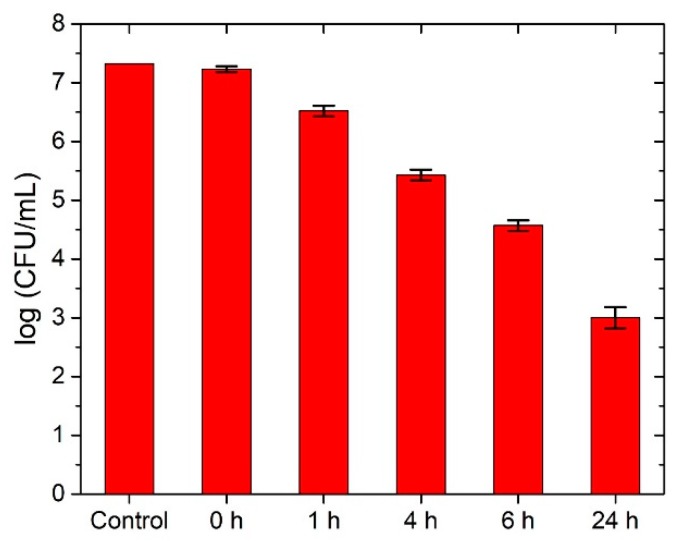
Antimicrobial test of AgNP-impregnated paper on *E. coli* . Please note the logarithmic scale on the y-axis.

These papers have been subjected to antibacterial tests using *E. coli* K12. As indicated in Figure 4, the impregnated materials are highly active toward *E. coli* K12. Figure 4 shows the AgNPs’ antimicrobial activity during 24 h, where it can be seen that the AgNPs’ antibacterial activity is increasing in time reaching up to a 4 log reduction rate.

**Figure 5:**
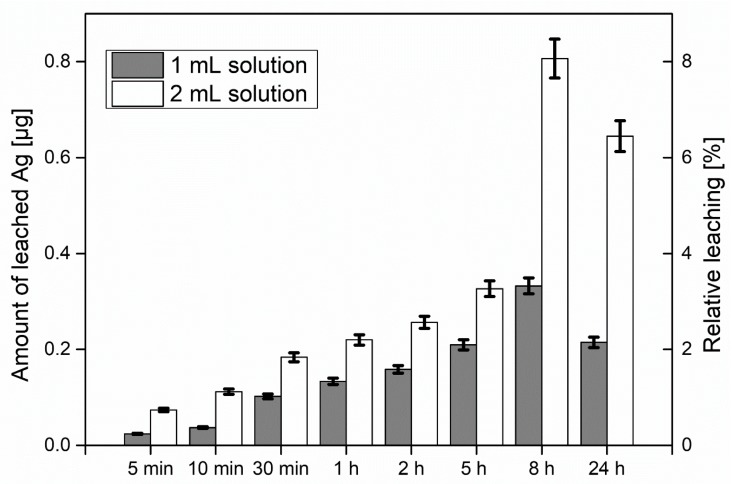
Amount of leached AgNPs from sheets and relative leaching with respect to the AgNPs used for the sheet formation. For the tests, 1 and 2 mL of the colloidal Ag dispersion (10 μg/mL Ag) were added to the fines suspension.

In order to investigate the leaching behavior of the AgNPs, the substrates were prepared with two different loadings of silver via the addition of 1 and 2 mL of the colloidal NP solution to the fines dispersion. The filtrate after formation of the sheets was colorless indicating that the AgNPs had been quantitatively incorporated into the sheets. Considering the total amount of AgNPs in the dispersion, this correlates to an AgNP content of 10 and 20 μg, respectively, in the sheets, which corresponds to 42 and 84 μg Ag per gram of paper. The relative mass of leaching AgNPs as well as the total leached mass of AgNPs after exposure to water under rigorous shaking are depicted in Figure 5. The amount of leached AgNPs was measured with ICP-MS after 5 min, 10 min, 30 min, 1 h, 2 h, 5 h, 8 h, and 24 h of substrate immersion in water and vigorous shaking. Relative leaching is determined with respect to the total amount of AgNPs in dispersion. It can be seen that the amount of leached AgNPs increases smoothly up to 8 h spent in immersion and under shaking conditions as expected. After 8 h the leaching is significantly increased for the denser solution, whereas after 24 h the leaching drops due to the degradation of the paper samples upon long exposure to water environment and shaking, points which will be discussed later.

## 4. Discussion

The use of LAL for the generation of AgNPs which are then implemented into a microstructured paper substrate is a step toward a scalable approach to implement these nanoparticles into cellulosic materials. There are several advantages of using this approach: the amount of applied nanoparticles can be precisely controlled, which is unlikely with any of the other methods. This is important in order to avoid overdosing and to equip materials with the required amount of nanoparticles to prevent bacterial growth, which is beneficial from both an ecological and an economic point of view. Almost any type of bulk material can be used to generate nanoparticles by LAL, and the use of reduction and capping agents can be avoided. In the case of AgNPs, a thin oxide layer results in a negatively charged surface (= −50 mV) making them stable in a colloidal solution according to the Derjagin-Landau-Verwey-Overbeek (DLVO) theory [45]. On the other hand, paper fines represent an underutilized stream in the paper industry and technology, exhibiting a few positive and several negative properties in the course of the paper manufacturing process. One of their drawbacks in paper production, namely their tendency to strongly interact with colloids, is exploited in the case of nanoparticles. After mixing the AgNPs with paper fines suspensions, the colored NPs are homogeneously distributed in the fines’ suspensions. Any precipitation of the AgNPs has not been observed, while aggregates on the fines surface are very rare. A certain interaction of these AgNPs with the fines must be occurring, because no removal of the AgNPs was observed during the sheet formation process, since the filtrates did not exhibit any color. In contrast, the sheets were slightly yellowish. 

The silver concentrations in the study’s system were rather small with only 10 AgNPs per m^2^. Nonetheless, the AgNP-decorated sheets exhibited antimicrobial activity which was the authors’ main goal. There were two types of particles: those that were in direct contact with the bacteria during testing and those that were buried inside the paper network, thereby slowly providing silver ions over time. The action mechanism of silver nanoparticles on bacteria is still not completely clear and fully understood, but the concentration, shape, and size of AgNPs are known to have a significant effect on inactivation effectiveness [46,47]. After the penetration of AgNPs inside the bacteria, NPs interact with intracellular materials, DNA loses its replication ability, and cellular proteins are inactivated [48]. 

Recent investigations into antimicrobial activity have indicated that bacterial growth is not suppressed by affecting the maximum growth rate but rather by extending the lag phase, that is, the time bacteria spend without replicating and adapting to a new environment. A continuous release of Ag^+^ ions exhibits higher antimicrobial effects than adding a specific amount of Ag^+^ at the beginning of the experiment [49]. Further, electrically generated Ag^+^ has better antimicrobial properties than other silver-based compounds [50,51,52]. 

Upon immersion in water under vigorous shaking, a small fraction of the AgNPs leached from the paper. The determined concentration increased slowly for a period of 8 h, whereas the leaching was directly correlated with the amount of particles in the paper. After 8 h of exposure to water, the paper network disassembled, leading to a burst of released silver into the solution. One might expect that these levels would rise for the 24 h sample, but the silver levels in the solution were smaller than after 8 h. Most likely, the AgNPs were readsorbing and reattaching into fines which were abraded from the paper substrates during the shaking process. 

## 5. Conclusions

The authors proposed a simple, fast, and environmentally friendly method to fabricate AgNP-impregnated paper fines sheets with antimicrobial activity. The method was based on the standard route for paper sheet formation to which a step was added—the addition of colloidal nanoparticles. The method worked for any type of colloidal nanoparticle solution synthesized by LAL, whereas a highly stable nanoparticle solution (high zeta-potential) was required for better dispersion into the paper solution and thus better homogeneity in the final product.

Further improvements in the antimicrobial activity will be assessed by optimizing the nanoparticle concentration and using different types of LAL nanoparticles. Future development of the method will be realized in terms of the application of a broad range of nanoparticles with unique properties (Cu, Ti, metal oxides, two-component) for different applications (catalysis, photoactivity, sensors).

## Supplementary Materials

The following are available online at http://www.mdpi.com/1996-1944/11/12/2412/s1.

## References

[1] Reishofer D., Rath T., Ehmann H.M., Gspan C., Dunst S., Amenitsch H., Plank H., Alonso B., Belamie E., Trimmel G. (2017). Biobased Cellulosic–CuInS_2_ Nanocomposites for Optoelectronic Applications. ACS Sustain. Chem. Eng..

[2] Breitwieser D., Kriechbaum M., Ehmann H.M.A., Monkowius U., Coseri S., Sacarescu L., Spirk S. (2015). Photoreductive generation of amorphous bismuth nanoparticles using polysaccharides-Bismuth-cellulose nanocomposites. Carbohydr. Polym..

[3] Breitwieser D., Spirk S., Fasl H., Ehmann H.M.A., Chemelli A., Reichel V.E., Gspan C., Stana-Kleinschek K., Ribitsch V. (2013). Design of simultaneous antimicrobial and anticoagulant surfaces based on nanoparticles and polysaccharides. J. Mat. Chem. B.

[4] Sahoo K., Biswas A., Nayak J. (2017). Effect of synthesis temperature on the UV sensing properties of ZnO-cellulose nanocomposite powder. Sens. Actuators A.

[5] Spiridonov V.V., Panova I.G., Makarova L.A., Afanasov M.I., Zezin S.B., Sybachin A.V., Yaroslavov A.A. (2017). The one-step synthesis of polymer-based magnetic γ-Fe_2_O_3_/carboxymethyl cellulose nanocomposites. Carbohydr. Polym..

[6] Van Rie J., Thielemans W. (2017). Cellulose–gold nanoparticle hybrid materials. Nanoscale.

[7] Croes S., Stobberingh E.E., Stevens K.N.J., Knetsch M.L.W., Koole L.H. (2011). Antimicrobial and Anti-Thrombogenic Features Combined in Hydrophilic Surface Coatings for Skin-Penetrating Catheters. Synergy of Co-Embedded Silver Particles and Heparin. ACS Appl. Mater. Interfaces.

[8] Shrivastava S., Bera T., Singh S.K., Singh G., Ramachandrarao P., Dash D. (2009). Characterization of Antiplatelet Properties of Silver Nanoparticles. ACS Nano.

[9] Moram S.S.B., Byram C., Shibu S.N., Chilukamarri B.M., Soma R.V. (2018). Ag/Au Nanoparticle-Loaded Paper-Based Versatile Surface-Enhanced Raman Spectroscopy Substrates for Multiple Explosives Detection. ACS Omega.

[10] Reishofer D., Ehmann H.M., Amenitsch H., Gspan C., Fischer R., Plank H., Trimmel G., Spirk S. (2017). On the formation of Bi_2_S_3_-cellulose nanocomposite films from bismuth xanthates and trimethylsilyl-cellulose. Carbohydr. Polym..

[11] Schlücker S. (2009). SERS Microscopy: Nanoparticle Probes and Biomedical Applications. Chem. Phys. Chem..

[12] Taajamaa L., Rojas O.J., Laine J., Yliniemi K., Kontturi E. (2013). Protein-assisted 2D assembly of gold nanoparticles on a polysaccharide surface. Chem. Commun..

[13] Zhicong M., Zilin G., Ruoxia C., Xiaoqing Y., Zhiqiang S., Wei G. (2018). Surface-Bioengineered Gold Nanoparticles for Biomedical Applications. Curr. Med. Chem..

[14] Coseri S., Spatareanu A., Sacarescu L., Rimbu C., Suteu D., Spirk S., Harabagiu V. (2015). Green synthesis of the silver nanoparticles mediated by pullulan and 6-carboxypullulan. Carbohydr. Polym..

[15] Donati I., Travan A., Pelillo C., Scarpa T., Coslovi A., Bonifacio A., Sergo V., Paoletti S. (2009). Polyol Synthesis of Silver Nanoparticles: Mechanism of Reduction by Alditol Bearing Polysaccharides. Biomacromolecules.

[16] Dahl J.A., Maddux B.L.S., Hutchison J.E. (2007). Toward Greener Nanosynthesis. Chem. Rev..

[17] Huang H., Yang X. (2004). Synthesis of polysaccharide-stabilized gold and silver nanoparticles: A green method. Carbohydr. Res..

[18] Breitwieser D., Moghaddam M.M., Spirk S., Baghbanzadeh M., Pivec T., Fasl H., Ribitsch V., Kappe C.O. (2013). In situ preparation of silver nanocomposites on cellulosic fibers—Microwave vs. conventional heating. Carbohydr. Polym..

[19] Jiang L.-P., Wang A.-N., Zhao Y., Zhang J.-R., Zhu J.-J. (2004). A novel route for the preparation of monodisperse silver nanoparticles via a pulsed sonoelectrochemical technique. Inorg. Chem. Commun..

[20] Ehmann H.M.A., Breitwieser D., Winter S., Gspan C., Koraimann G., Maver U., Sega M., Köstler S., Stana-Kleinschek K., Spirk S., Ribitsch V. (2015). Gold nanoparticles in the engineering of antibacterial and anticoagulant surfaces. Carbohydr. Polym..

[21] Yan G. (2012). Laser Ablation in Liquids: Principles and Applications in the Preparation of Nanomaterials.

[22] Barcikowski S., Mafuné F. (2011). Trends and Current Topics in the Field of Laser Ablation and Nanoparticle Generation in Liquids. J. Phys. Chem. C.

[23] Kang H.W., Lee H., Welch A.J. (2008). Laser ablation in liquid confinement using a nanosecond laser pulse. J. Appl. Phys..

[24] Zhang D., Gökce B., Barcikowski S. (2017). Laser Synthesis and Processing of Colloids: Fundamentals and Applications. Chem. Rev..

[25] Besner S., Kabashin A.V., Winnik F.M., Meunier M. (2008). Ultrafast laser based ‘green’ synthesis of non-toxic nanoparticles in aqueous solutions. Appl. Phys. A.

[26] Dolgaev S.I., Simakin A.V., Voronov V.V., Shafeev G.A., Bozon-Verduraz F. (2002). Nanoparticles produced by laser ablation of solids in liquid environment. Appl. Surf. Sci..

[27] Tarasenko N.V., Butsen A.V. (2010). Laser synthesis and modification of composite nanoparticles in liquids. Quantum Electron..

[28] Tsuji T., Watanabe N., Tsuji M. (2003). Laser induced morphology change of silver colloids: Formation of nano-size wires. Appl. Surf. Sci..

[29] Tsuji T., Okazaki Y., Higuchi T., Tsuji M. (2006). Laser-induced morphology changes of silver colloids prepared by laser ablation in water Enhancement of anisotropic shape conversions by chloride ions. J. Photochem. Photobiol. A.

[30] Zeng H., Yang S., Cai W. (2011). Reshaping Formation and Luminescence Evolution of ZnO Quantum Dots by Laser-Induced Fragmentation in Liquid. J. Phys. Chem. C.

[31] Giorgetti E., Giammanco F., Marsili P., Giusti A. (2011). Effect of Picosecond Postirradiation on Colloidal Suspensions of Differently Capped AuNPs. J. Phys. Chem. C.

[32] Burakov V.S., Tarasenko N.V., Butsen A.V., Rozantsev V.A., Nedel’ko M.I. (2005). Formation of nanoparticles during double-pulse laser ablation of metals in liquids. Eur. Phys. J. Appl. Phys..

[33] DeGiacomo A., DeBonis A., Dell’Aglio M., De Pascale O., Gaudiuso R., Orlando S., Santagata A., Senesi G.S., Taccogna F., Teghil R. (2011). Laser Ablation of Graphite in Water in a Range of Pressure from 1 to 146 atm Using Single and Double Pulse Techniques for the Production of Carbon Nanostructures. J. Phys. Chem. C.

[34] Dell’Aglio M., Gaudiuso R., El Rashedy R., De Pascale O., Palazzo G., De Giacomo A. (2013). Collinear double pulse laser ablation in water for the production of silver nanoparticles. Phys. Chem. Chem. Phys..

[35] Krstulovic N., Shannon S., Stefanuik R., Fanara C. (2013). Underwater-laser drilling of aluminum. Int. J. Adv. Manuf. Technol..

[36] Krstulovic N., Umek P., Salamon K., Capan I. (2017). Synthesis of Al-doped ZnO nanoparticles by laser ablation of ZnO:Al_2_O_3_ target in water. Mater. Res. Express.

[37] Krstulovic N., Salamon K., Budimlija O., Kovac J., Dasovic J., Umek P., Capan I. (2018). Parameters optimization for synthesis of Al-doped ZnO nanoparticles by laser ablation in water. Appl. Surf. Sci..

[38] Krstulovic N., Milosevic S. (2010). Drilling enhancement by nanosecond–nanosecond collinear dual-pulse laser ablation of titanium in vacuum. Appl. Surf. Sci..

[39] Fischer W.J., Mayr M., Spirk S., Reishofer D., Jagiello L.A., Schmiedt R., Colson J., Zankel A., Bauer W. (2017). Pulp Fines—Characterization, Sheet Formation, and Comparison to Microfibrillated Cellulose. Polymers.

[40] Kiss L.B., Söderlund J., Niklasson G.A., Granqvist C.G. (1999). New approach to the origin of lognormal size distributions of nanoparticles. Nanotechnology.

[41] Söderlund J., Kiss L.B., Niklasson G.A., Granqvist C.G. (1998). Lognormal size distributions in particle growth processes without coagulation. Phys. Rev. Lett..

[42] Mafuné F., Kohno J., Takeda Y., Kondow T. (2000). Formation and Size Control of Silver Nanoparticles by Laser Ablation in Aqueous Solution. J. Phys. Chem. B.

[43] Zeng H., Du X.-W., Singh S.C., Kulinich S.A., Yang S., He J., Cai W. (2012). Nanomaterials via Laser Ablation/Irradiation in Liquid: A Review. Adv. Funct. Mater..

[44] Zhang D.S., Liu J., Liang C.H. (2017). Perspective on how laser-ablated particles grow in liquids. Sci. China-Phys. Mech. Astron..

[45] Lyklema J., van Leeuwen H.P., Minor M. (1999). DLVO-theory, a dynamic re-interpretation. Adv. Colloid Interface Sci..

[46] Agnihotri S., Mukherji S., Mukherji S. (2014). Size-controlled silver nanoparticles synthesized over the range 5–100 nm using the same protocol and their antibacterial efficacy. RSC Adv..

[47] Sondi I., Salopek-Sondi B. (2004). Silver nanoparticles as antimicrobial agent: A case study on E. coli as a model for Gram-negative bacteria. J. Colloid Interface Sci..

[48] Morones J.R., Elechiguerra J.L., Camacho A., Holt K., Kouri J.B., Ramírez J.T., Yacaman M.J. (2005). The bactericidal effect of silver nanoparticles. Nanotechnology.

[49] Liau S., Read D., Pugh W., Furr J., Russell A. (1997). Interaction of silver nitrate with readily identifiable groups: Relationship to the antibacterialaction of silver ions. Lett. Appl. Microbiol..

[50] Berger T.J., Spadaro J.A., Bierman R., Chapin S.E., Becker R.O. (1976). Antifungal properties of electrically generated metallic ions. Antimicrob. Agents Chemother..

[51] Berger T.J., Spadaro J.A., Chapin S.E., Becker R.O. (1976). Electrically generated silver ions: Quantitative effects on bacterial and mammalian cells. Antimicrob. Agents Chemother..

[52] Vukusic T., Shi M., Herceg Z., Rogers S., Estifaee P., Mededovic Thagard S. (2016). Liquid-phase electrical discharge plasmas with a silver electrode for inactivation of a pure culture of Escherichia coli in water. Innov. Food Sci. Emerg. Technol..

