# Supplementary figures and images for: Green Procedure to Manufacture Nanoparticle-Decorated Paper Substrates
Source: Materials (Basel). 2018 Nov 29;11(12):2412. doi: 10.3390/ma11122412 (PMC6316935; doi:10.3390/ma11122412)

## EDS analysis

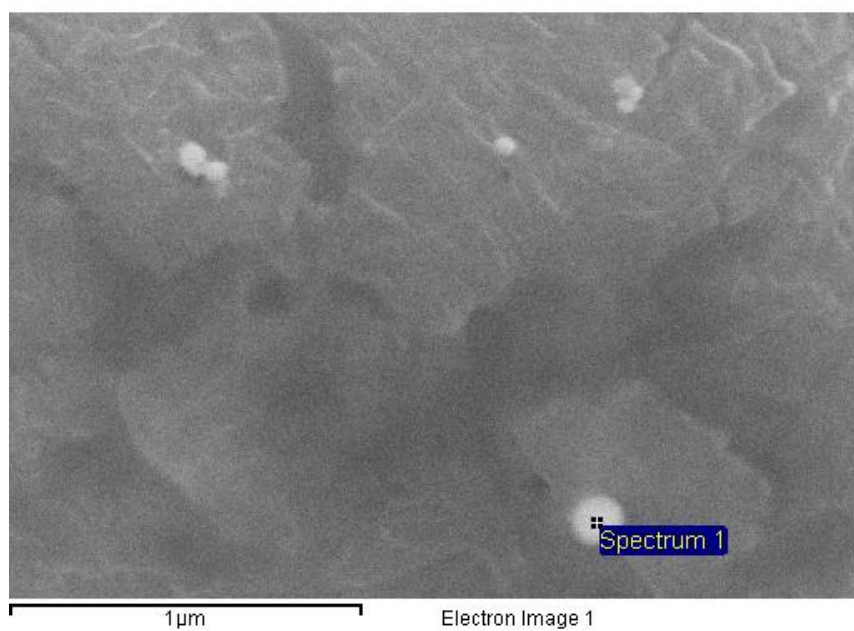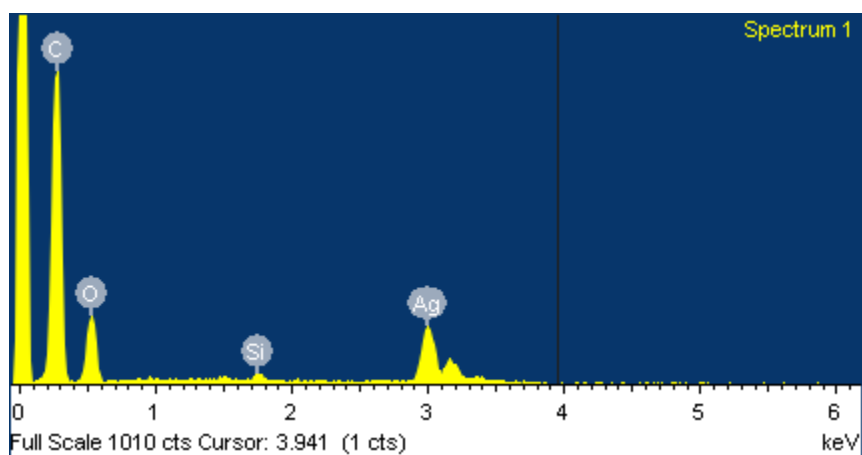

| Element | Weight (%) | Atomic (%) |
|---------|------------|------------|
| C K     | 51.00      | 67.34      |
| O K     | 29.85      | 29.59      |
| Si K    | 0.60       | 0.34       |
| Ag L    | 18.55      | 2.73       |
| TOTAL   | 100        |            |

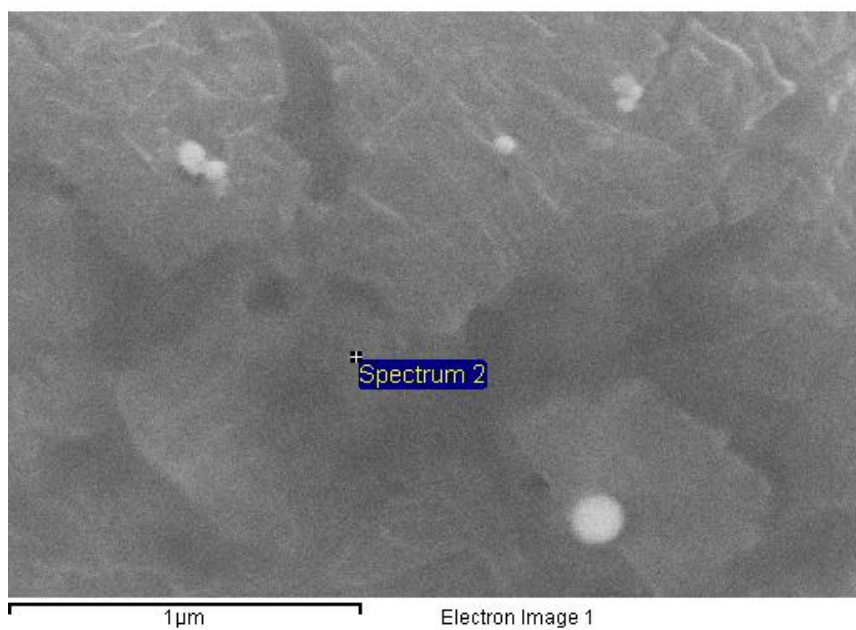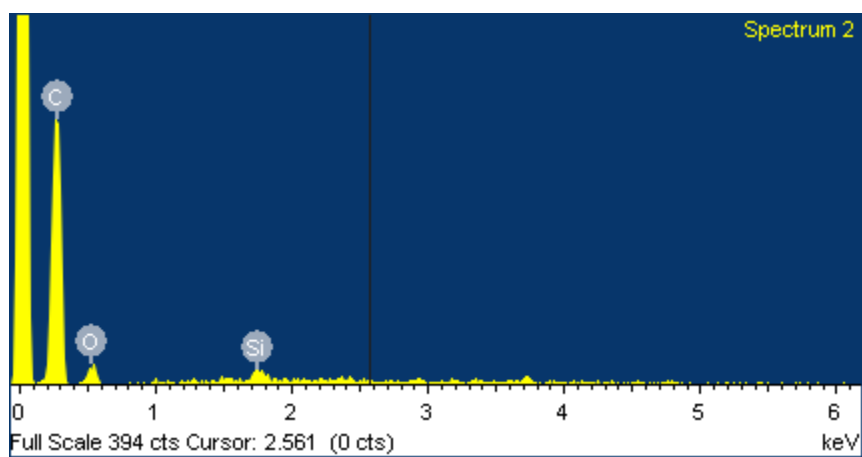

| Element | Weight (%) | Atomic (%) |
|---------|------------|------------|
| C K     | 82.29      | 86.54      |
| O K     | 16.16      | 12.76      |
| Si K    | 1.54       | 0.69       |
| TOTAL   | 100        |            |

Supplement: Supplementary file 1 [file materials-11-02412-s001.pdf]
